# Sociodemographic changes and trends in the rates of new perinatal HIV diagnoses and transmission in Spain from 1997 to 2015

**DOI:** 10.1371/journal.pone.0223536

**Published:** 2019-10-24

**Authors:** Santiago Jiménez de Ory, José Tomas Ramos, Claudia Fortuny, María Isabel González-Tomé, Maria José Mellado, David Moreno, César Gavilán, Ana Isabel Menasalvas, Ana Isabel Piqueras, M. Antoinette Frick, Maria Angeles Muñoz-Fernández, Maria Luisa Navarro, on behalf of the CoRISpe Cohort Working Group

**Affiliations:** 1 Hospital General Universitario Gregorio Marañón, Instituto de Investigación Sanitaria Gregorio Marañón (IisGM), CoRISpe, Madrid, Spain; 2 Servicio de Pediatría, Hospital Clínico San Carlos, Universidad Complutense de Madrid, Instituto de Investigación Sanitaria del Hospital Clínico San Carlos (IdISSC), Madrid, Spain; 3 Unidad de Enfermedades Infecciosas, Servicio de Pediatría, Hospital Sant Joan de Déu, Universitat de Barcelona, Esplugues del Llobregat, Spain; 4 Servicio de Infecciosas Pediátricas, Hospital Universitario Doce de Octubre, Instituto de Investigación Hospital 12 de Octubre, Universidad Complutense de Madrid, Madrid, Spain; 5 Pediatrics, Immunodeficiencies and Infectious Diseases Unit, Hospital Universitario La Paz, Madrid, Spain; 6 Translational Research Network in Pediatric Infectious Diseases (RITIP), Madrid, Spain; 7 Department of Pediatrics, Regional Maternal-Child University Hospital, Malaga, Spain; 8 IBIMA Multidisciplinary Group for Pediatric Research, Malaga, Spain, Malaga University, Malaga, Spain; 9 Department of Paediatrics, University Clinical Hospital of San Juan de Alicante, San Juan de Alicante, Alicante, Spain; 10 Department of Paediatrics, Miguel Hernández University of Elche, Campus of Sant Joan d'Alacant, Alicante, Spain; 11 Department of Paediatrics, Hospital Virgen de la Arrixaca, Murcia, España; 12 Department of Pediatric Surgery, and Department of Pediatrics, Hospital La Fe, Valencia, Spain; 13 Tropical Medicine and International Health Unit. Hospital Universitari Vall d'Hebron, Barcelona, Spain; 14 Department of Pediatrics, Hospital Universitari Vall d'Hebron, Barcelona, Spain; 15 PROSICS Barcelona, Universitat Autònoma de Barcelona, Barcelona, Spain; 16 Section Immunology, Laboratorio InmunoBiología Molecular, Hospital General Universitario Gregorio Marañón, Instituto de Investigación Sanitaria Gregorio Marañón, Madrid, Spain; 17 Networking Research Center on Bioengineering, Biomaterials and Nanomedicine (CIBER-BBN), Spain, Spanish HIV HGM BioBank, Madrid, Spain; 18 Sección de Enfermedades Infecciosas, Servicio de Pediatría, Hospital General Universitario Gregorio Marañón and Instituto de Investigación Sanitaria Gregorio Marañón, Medical School, Universidad Complutense de Madrid, Translational Research Network in Pediatric Infectious Diseases (RITIP), Madrid, Spain; University of Cyprus, CYPRUS

## Abstract

**Background:**

There are not enough nationwide studies on perinatal HIV transmission in connection with a combination of antiretroviral treatments in Spain. Our objectives were to study sociodemographic changes and trends in the rates of HIV diagnoses and perinatal transmission in Spain from 1997 to 2015.

**Methods:**

A retrospective study using data from Spanish Paediatric HIV Network (CoRISpe) and Spanish Minimum Basic Data Set (MDBS) was performed. HIV- diagnosed children between 1997 and 2015 were selected. Sociodemographic, clinical and immunovirological data of HIV-infected children and their mothers were studied in four calendar periods (P1: 1997–2000; P2: 2001–2005; P3: 2006–2010; P4: 2011–2015). Rates of perinatal HIV diagnoses and transmission from 1997 to 2015 were calculated.

**Results:**

A total of 532 HIV-infected children were included in this study. Of these children, 406 were Spanish (76.3%) and 126 immigrants (23.7%). A decrease in the number of HIV diagnoses, 203 (38.2%) children in the first (P1), 149 (28%) in the second (P2), 130 (24.4%) in the third (P3) and 50 (9.4%) in the fourth (P4) calendar periods was studied. The same decrease in the Spanish HIV-infected children (P1, 174 (46.6%), P2, 115 (30.8%), P3, 65 (17.4%) and P4, 19 (5.1%)) was monitored. However, an increase in the number of HIV diagnoses by sexual contact (P1: 0%; P2: 1.3%; P3: 4.6%; P4: 16%) was observed. The rates of new perinatal HIV diagnoses and perinatal transmission in Spanish children decreased from 0.167 to 0.005 per 100,000 inhabitants and 11.4% to 0.4% between 1997 and 2015, respectively.

**Conclusions:**

A decline of perinatal HIV diagnoses and transmission was observed. However, an increase of teen-agers HIV diagnoses with sexual infection was studied. Public awareness campaigns directed to teen-agers are advisable to prevent HIV infection by sexual contact.

## Introduction

Perinatal HIV transmission has decreased to below 2% in newborns in high-income countries (HIC) due to the prevention of mother-to-child transmission (PMTCT) and combination antiretroviral therapy (cART) [1–4]. However, information on sociodemographic changes and trends in the rates of perinatal HIV transmission in Spain in the cART era has been obtained from local cohorts [5–9], but not from a nationwide perspective. Although Spanish Information Systems on New HIV Diagnoses (SINIVIH) include epidemiological, clinical and immunological data on new HIV diagnoses since the year 2000, the implementation of the SINIVIH was progressive, and did not cover all the Spanish population until the year 2013 [10].

Our objectives were: 1) to study sociodemographic changes in HIV-infected children and teen-agers who were diagnosed in Spain; 2) to study sociodemographic changes in perinatally HIV-infected children born in Spain and in their mothers; 3) to estimate the rate of new perinatal HIV diagnoses in children born in Spain; 4) to estimate the rate of perinatal HIV transmission in Spain from 1997 to 2015.

## Materials and methods

### Data sources

#### The Spanish Pediatric HIV Network (CoRISpe)

The Spanish Pediatric HIV Network (CoRISpe), which collaborates actively with the Spanish HIV HGM BioBank, is an open, multicenter, retrospective, and prospective cohort founded in 2008 in accordance with Spanish law on the protection of personal data [11–12]. CoRISpe collects epidemiological, clinical, immunological, virological, analytical, and antiretroviral retrospective data from HIV-infected children and teen-agers since 1995 and the same prospective data from HIV-infected children and teen-agers between the age of 0 and 18 years, with follow-up in Spanish pediatric HIV units (SPHU) since 2008. At 31 December 2017, data from 1335 HIV-infected children and teen-agers coming from 63 Spanish hospitals, belonging to 17 Autonomous Communities, were collected in CoRISpe. Relevant results from those data on characteristics and trends of new HIV diagnoses in Spain were obtained [13].

#### Spanish minimum basic data set

The Minimum Basic Data Set (MBDS) of the National Surveillance System for Hospital Data in Spain, provided by the Ministry of Health, Consumer Affairs and Social Welfare (MSSSI), is a clinical and administrative database containing clinical information recorded at the time of hospital discharge. The MBDS has an estimated coverage of 97.7% of total public hospital admissions. This database provides encrypted patient identification numbers, gender, date of birth, postal codes of patient’s place, dates of hospital admission and discharge, medical institutions providing the services, the diagnosis and procedure codes according to the International Classification of Diseases 9^th^ Revision, Clinical Modification (ICD-9-CM), as well as the outcome at discharge [14–15].

### Ethics statement

The study was conducted according to the Declaration of Helsinki and was approved by the Ethical Committees of each participating hospital of the CoRISpe working group, including the Ethics and Clinical Research Committee of País Vasco, Aragón, Navarra, La Rioja, Galicia, Granada, Huelva, Hospital Universitario La Paz, Hospital Universitario Gregorio Marañón, Hospital Universitario de Getafe, Hospital Universitario Doce de Octubre, Hospital Universitario de Mostoles, Hospital Universitario Príncipe de Asturias, Hospital de Torrejón, Hospital Clinico San Carlos Complejo Hospitalario de Toledo, Complejo Hospitalario de Albacete Hospital Marques de Valdecilla, Hospital Universitari Vall d'Hebron, Hospital Universitario Central de Asturias, Hospital Universitario Nuestra Señora de la Candelaria, Complejo Hospitalario Universitario Insular-Materno Infantil, Infanta Cristina de Badajoz, Complejo Hospitalario de Cáceres, Hospital Universitario Virgen de la Arrixaca, Hospital Clínico Universitario de Valencia, Hospital Universitario La Fe, Hospital General de Castelló, Hospital San Juan de Alicante, Área de Salud de Zamora, Hospital Clínico Universitario de Valladolid, Complejo Asistencial de León, Hospital Regional Universitario Carlos Haya, Complejo Hospitalario de Torrecárdenas, Hospital de Poniente, Hospital Universitario Virgen de las Nieves, Hospital Universitario Virgen de la Macarena and Hospital Universitario Virgen del Rocío. Written informed consent was obtained from all children’s parents/guardians, as well as from all participants above 12 years old. Respect to the MDBS, the MSSSI evaluated the protocol of our study and considered that it fulfilled all ethical considerations according to the Spanish legislation. The data were treated with full confidentiality according to the Spanish legislation.

### Children and data selection

HIV-infected children included in the CoRISpe who were diagnosed in Spain from 1st January 1997 to 31st December 2015 were included in the study. HIV-infected children were excluded if they were diagnosed in foreign countries or if the children were first followed in SPHU not participating in the CoRISpe.

#### 1) Sociodemographic changes in HIV-infected children

At the time of HIV diagnosis the collected data were sociodemographic (birth country, sex, HIV transmission and birth date), clinical (Centers for Diseases Control and Prevention (CDC) stage: N-A: not or mildly symptomatic; B: moderately symptomatic; C: severely symptomatic), comorbidity (HCV and HBV infections), immunological (CD4^+^ T lymphocyte count (CD4/mm^3^) and CD4+ T-lymphocyte percent of total lymphocytes (%CD4)) and virological (viral load; (VL)) data in copies/mL. Immunological categories based on %CD4 were used: category 1, no damage (25% or over); category 2, moderate (15–24%); category 3, severe (less than 15%) [16]. According to the year of HIV diagnosis four calendar periods were considered: period 1 (P1) from 1997 to 2000; period 2 (P2) from 2001 to 2005; period 3 (P3) from 2006 to 2010; period 4 (P4) from 2011 to 2015.

#### 2) Sociodemographic changes in perinatally HIV-infected children born in Spain and their mothers

Perinatally HIV-infected children born in Spain were selected. At HIV diagnosis, sociodemographic (sex, birth and diagnosis date), clinical (CDC stage, with the same categories mentioned above: N-A: not or mildly symptomatic; B: moderately symptomatic; C: severely symptomatic), comorbidity (HCV and HBV infections), immunological (CD4/mm^3^ and %CD4) and virological (VL) data of children were collected. The same immunological categories based on %CD4 mentioned above were used: category 1, no damage (25% or over); category 2, moderate (15–24%); category 3, severe (less than 15%) [16]. According to the age of HIV diagnosis, children were classified in three groups: 1) <1 year, if they were diagnosed with <1 year of age; 2) 1–5 years, if they were diagnosed between 1 and 5 years of age; 3) >5 years, if they were diagnosed with >5 years of age. The following data were collected from their mothers: birth country, mode of HIV transmission and period of HIV diagnosis. According to the period of HIV diagnosis, mothers were classified in two groups: 1) until childbirth, if they were diagnosed before, during pregnancy or at childbirth; 2) after childbirth, if they were diagnosed after childbirth.

According to the mode of HIV transmission, mothers were classified as: 1) intravenous drug users (IDU), if they took intravenous drugs before or during pregnancy, and 2) No history of IDU, if they did not take them before or during pregnancy. The same four calendar periods, mentioned above, were considered for the analysis, according to HIV diagnosis year of the children (P1: 1997–2000; P2: 2001–2005; P3: 2006–2010; P4: 2011–2015).

#### 3) Estimating the rate of new perinatal HIV diagnoses in children born in Spain

Perinatally HIV-infected children born in Spain were selected. Population projections in Spain were obtained from the National Statistics Institute on the first day of December every year to calculate rates, using the population of each year as the denominator [17]. Population of provinces with no SPHU participating in the CoRISpe was excluded from the denominator. Population of provinces with some SPHU participating in the CoRISpe, but not all, was adjusted according to the proportion of HIV-infected children followed in those SPHU which participate in the CoRISpe. The total rate of new HIV diagnoses from 1997 to 2015 was calculated by dividing the number of perinatally HIV-infected children by the sum of the corresponding population in the study period (i.e., number of cases/100,000 inhabitants).

#### 4) Estimating the rate of perinatal HIV transmission in Spain

All hospitalizations between 1997 and 2015 that were coded in the MBDS with a childbirth procedure were reviewed. The ICD-9-CM codes for defining childbirths were used: 72 (forceps, vacuum and breech delivery); 73 (other procedures inducing or assisting delivery); 74 (cesarean section and removal of fetus). Postal codes of patient’s place of residence to adjust the number of childbirths were used if these childbirths took place in the provinces with not SPHU participating in the CoRISpe, or to adjust the number of childbirths, if these childbirths took place in the provinces with some SPHU participating in the CoRISpe. ICD-9-CM codes 042 (HIV disease) and V08 (asymptomatic HIV infection status) for defining HIV infection in the mothers were used [15]. The percentage of childbirths from HIV-infected mothers that respect to total childbirths, per year, was calculated by dividing the number of childbirths from HIV infected mothers by the total childbirths. Perinatally HIV-infected children born in Spain from 1st January 1997 to 31st December 2015, whose mothers were HIV-diagnosed until childbirth, were selected from the children included in our study to estimate the rate of perinatal HIV transmission. The rate of perinatal HIV transmission, per year, was estimated by dividing the number of perinatally HIV-infected children by the number of childbirths from HIV-infected mothers.

### Statistical analysis

Results involving categorical variables were expressed as proportions, whereas results involving continuous variables were expressed as medians and interquartile ranges.

## Results

### 1) Sociodemographic changes in HIV-infected children

**Fig 1:**
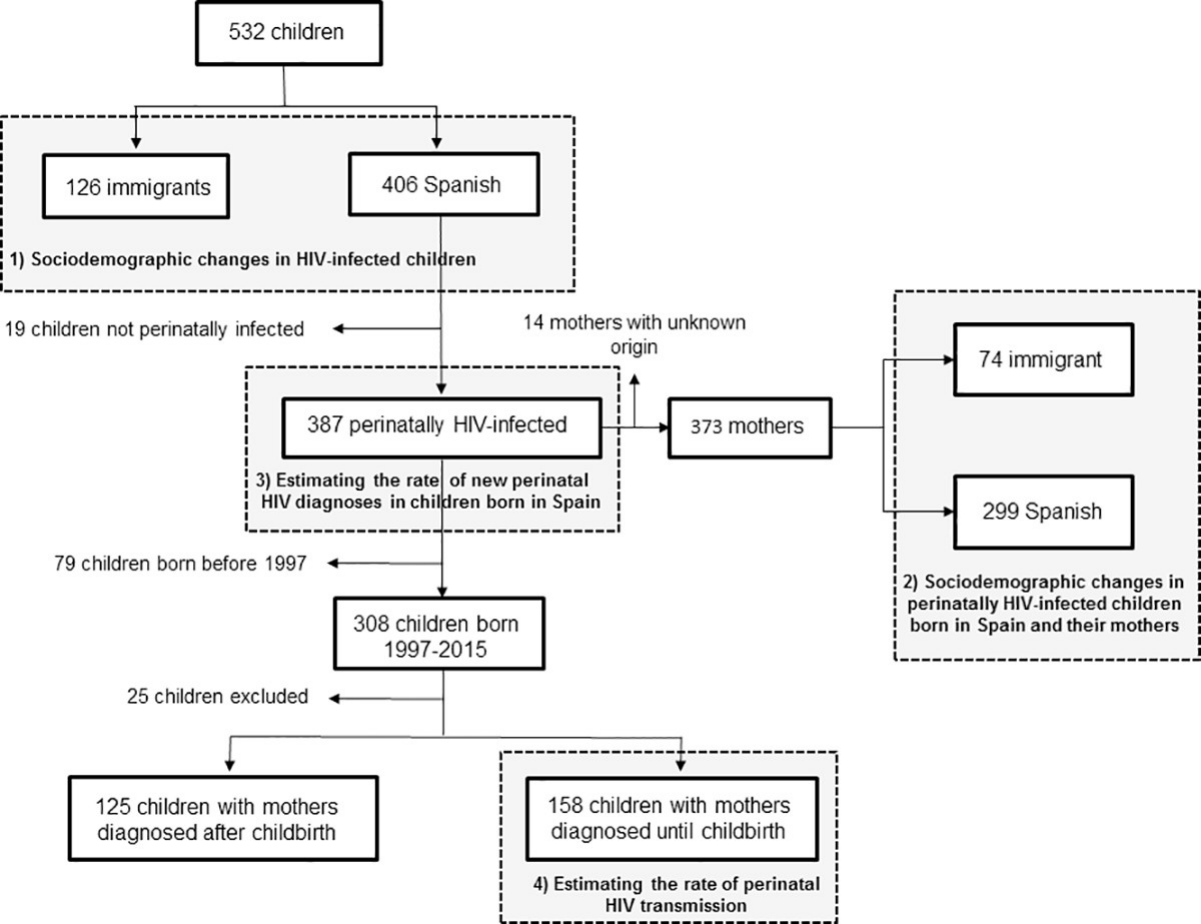
Flow-chart for the selection of HIV-infected children diagnosed in Spain from 1997 to 2015.

A total of 624 new cases of HIV-infected children and teen-ages in the CoRISpe were observed from 1997 to 2015. Out of them, 77 children were excluded from this study because they were not diagnosed with HIV infection in Spain. Fifteen children were excluded because they were first followed in SPHU not participating in the CoRISpe. Of the remaining 532 children, 406 were Spanish (76.3%) and 126 were immigrants (23.7%) (Fig 1).

All children, except one HIV-2 infected child through blood products transfusion from sub-Saharan Africa, were HIV-1 infected. Immigrant children came mainly from sub-Saharan Africa (82 out of 126 (65.1%)) and Latin America (27 out of 126 (21.4%)). Children born in foreign countries had lower %CD4 and CD4/mm^3^ and were in worse clinical situation at diagnosis than children born in Spain. A decrease in the number of HIV diagnoses was observed through the calendar periods, mainly in Spanish children, whereas the number of immigrant children increased from P1 to P3 and decreased in P4 (Table 1).

**Table 1:** Sociodemographic, clinical, immunological and virological profile of the HIV-infected children at diagnosis, by origin of the children.

	Spanish	Immigrants
	N = 406	N = 126
**Calendar period, N (%)**		
1997–2000	191 (47)	12 (9.5)
2001–2005	118 (29.1)	31 (24.6)
2006–2010	73 (18)	57 (45.2)
2011–2015	24 (5.9)	26 (20.6)
**Sex, N (%)**		
Male	187 (46.1)	64 (50.8)
Female	219 (53.9)	62 (49.2)
**HIV transmission mode, N (%)**		
Perinatal	387 (95.3)	89 (70.6)
Transfusional	3 (0.7)	11 (8.7)
Sexual	6 (1.5)	10 (7.9)
Unknown (mother not HIV infected)	10 (2.5)	2 (1.6)
Unknown (HIV mother status not known)	0 (0)	14 (11.1)
**CDC stage, N (%)**	N = 402	N = 125
N-A	267 (66.4)	61 (48.8)
B	78 (19.4)	31 (24.8)
C	57 (14.2)	33 (26.4)
**Coinfections, N (%)**		
HCV	12 (3)	1 (0.8)
HBV	0 (0)	9 (7.1)
**%CD4, Median (IQR)**	N = 346	N = 119
	28 (15.2–40)	17 (8.3–24.7)
**CD4/mm**^**3**^**, Median (IQR)**	N = 348	N = 119
	1176 (465–2185)	547 (213–1036)
**Log Viral Load, Median (IQR)**	N = 354	N = 118
	5.2 (4.6–5.9)	5.1 (4.5–5.7)

Thirteen children were HCV-coinfected, all were Spanish except one child from sub-Saharan Africa. Nine children were HBV-coinfected, all were from sub-Saharan Africa except one child from Eastern Europe (Table 1). Sixteen teen-agers, 8 male and 8 female, were HIV-infected by sexual contact. They were mainly from Latin America (8 out of 16 (50%)) and Spain (6 out of 16 (37.5%)), with median age 16.4 years [IQR: 15.4–16.7], 26%CD4 [IQR: 20.5–31] and 510 CD4/mm^3^ [IQR: 394–631] at diagnosis. Twelve out of 16 teen-agers (75%) had CDC stage A, 2 out 16 (12.5%) had CDC stage B and 2 out of 16 (12.5%) had CDC stage C at diagnosis. Three out of 16 (18.8%) had <350 CD4/mm^3^, two of them had <200 CD4/mm^3^.

A slight increase in sexual HIV diagnoses was observed throughout the study period (P1: 0 out of 203 (0%); P2: 2 out of 149 (1.3%); P3: 6 out of 130 (4.6%); P4: 8 out of 50 (16%)). See S1 Table for complete data by origin of the children.

### 2) Sociodemographic changes in perinatally HIV-infected children born in Spain and their mothers

Out of 406 Spanish children, 387 (95.3%) were perinatally HIV-infected. Origin of 373 mothers could be obtained, 299 were Spanish (80.2%) and 74 immigrants (19.8%) (Fig 1). Immigrant mothers were mainly from sub-Saharan Africa (39 out of 74 (52.7%)) and Latin America (19 out of 74 (25.7%)). A decrease in the number of HIV-diagnosed children throughout the study period was observed. This decrease was observed mainly in children born from Spanish mothers, whereas the number of HIV-diagnosed children born from immigrant mothers increased from P1 to P3, but declined in P4. There was a higher percentage of Spanish IDU mothers than immigrant IDU mothers (52% vs. 5.6%, respectively). A decrease in the number of IDU mothers was observed through the calendar periods (Table 2).

**Table 2:** Sociodemographic, clinical, immunological and virological profile at diagnosis of perinatally HIV-infected children born in Spain and their mothers, by calendar periods.

	1997–2000	2001–2005	2006–2010	2011–2015
	N = 174	N = 115	N = 65	N = 19
**Origin of mothers, N (%)**				
Spanish	163 (93.7)	95 (82.6)	32 (49.2)	9 (47.4)
Immigrants	11 (6.3)	20 (17.4)	33 (50.8)	10 (52.6)
**Period of HIV diagnosis for mothers, N (%)**				
Until childbirth	89 (51.1)	47 (40.9)	36 (55.4)	10 (52.6)
After childbirth	85 (48.9)	68 (59.1)	29 (44.6)	9 (47.4)
**Mode of HIV transmission of mothers, N (%)**	N = 163	N = 110	N = 61	N = 18
IDU	90 (55.2)	47 (42.7)	11 (18)	2 (11.1)
No history of IDU	73 (44.8)	63 (57.3)	50 (82)	16 (88.9)
**Sex of children, N (%)**				
Male	71 (40.8)	60 (52.2)	29 (44.6)	8 (42.1)
Female	103 (59.2)	55 (47.8)	36 (55.4)	11 (57.9)
**Age of children, N (%)**				
<1 year	109 (62.6)	74 (64.3)	45 (69.2)	13 (68.4)
1–5 years	51 (29.3)	25 (21.7)	15 (23.1)	4 (21.1)
>5 years	14 (8)	16 (13.9)	5 (7.7)	2 (10.5)
**Coinfections in children, N (%)**				
HCV	9 (5.2)	2 (1.7)	0 (0)	0 (0)
HBV	0 (0)	0 (0)	0 (0)	0 (0)
**CDC stage of children, N (%)**	N = 169	N = 113	N = 65	N = 19
N-A	87 (51.4)	71 (62.8)	47 (72.3)	17 (89.5)
B	41 (24.3)	26 (23)	9 (13.8)	1 (5.3)
C	41 (24.3)	16 (14.2)	9 (13.8)	1 (5.3)
**%CD4, Median (IQR)**	N = 148	N = 99	N = 63	N = 19
	27.4 (16–36)	27 (13.9–40.5)	31.7 (21–46)	36 (27.5–44.9)
**CD4/mm**^**3**^**, Median (IQR)**	N = 149	N = 99	N = 63	N = 19
	1192 (439–2126)	1145 (578–2126)	1775 (691–2963)	1489 (935–2567)
**Log Viral Load, Median (IQR)**	N = 153	N = 99	N = 63	N = 18
	5.5 (4.9–6.1)	5.2 (4.7–5.7)	5.2 (4.2–5.9)	5.1 (4.5–5.7)

Eleven children were HCV-coinfected, all born from Spanish IDU mothers except one child born from a Latin America mother, who was HIV-infected by sexual contact. A decrease in the number of HCV-coinfected children was observed through the calendar periods, with no cases in P3 and P4. Regarding the clinical and immune situation, a decrease in the number of children with CDC B and C stages was observed throughout the study period, as well as a slight increase in %CD4 (Table 2). See S2 Table for complete data by origin of the mothers.

### 3) Estimating the rate of new perinatal HIV diagnoses in children born in Spain

**Fig 2:**
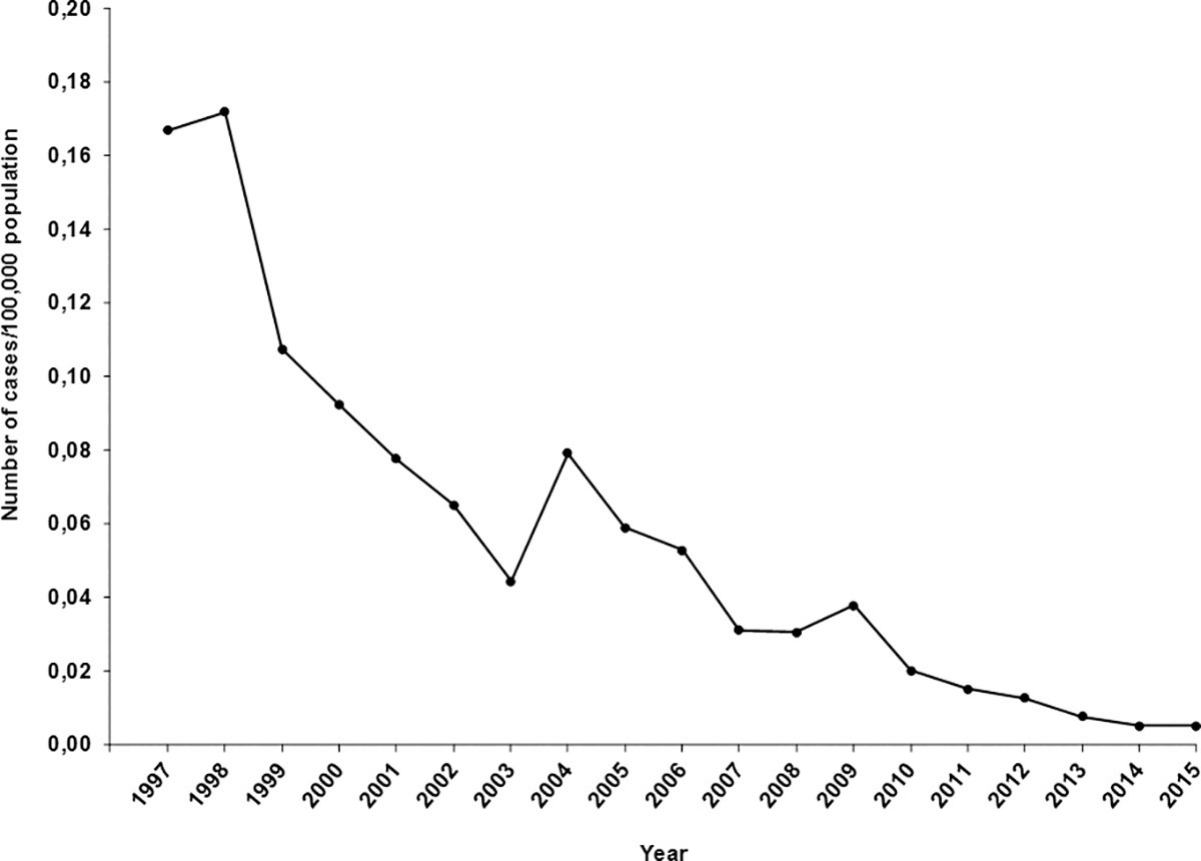
Rate of new perinatal HIV diagnoses in children born in Spain from 1997 to 2015.

Out of 406 Spanish children, 387 perinatally HIV-infected children were selected (Fig 1). A decrease in the rate of new diagnoses by perinatal transmission per 100,000 inhabitants was observed, from 0.167 in 1997 to 0.005 in 2015 (Fig 2).

### 4) Estimating the rate of perinatal HIV transmission in Spain

**Fig 3:**
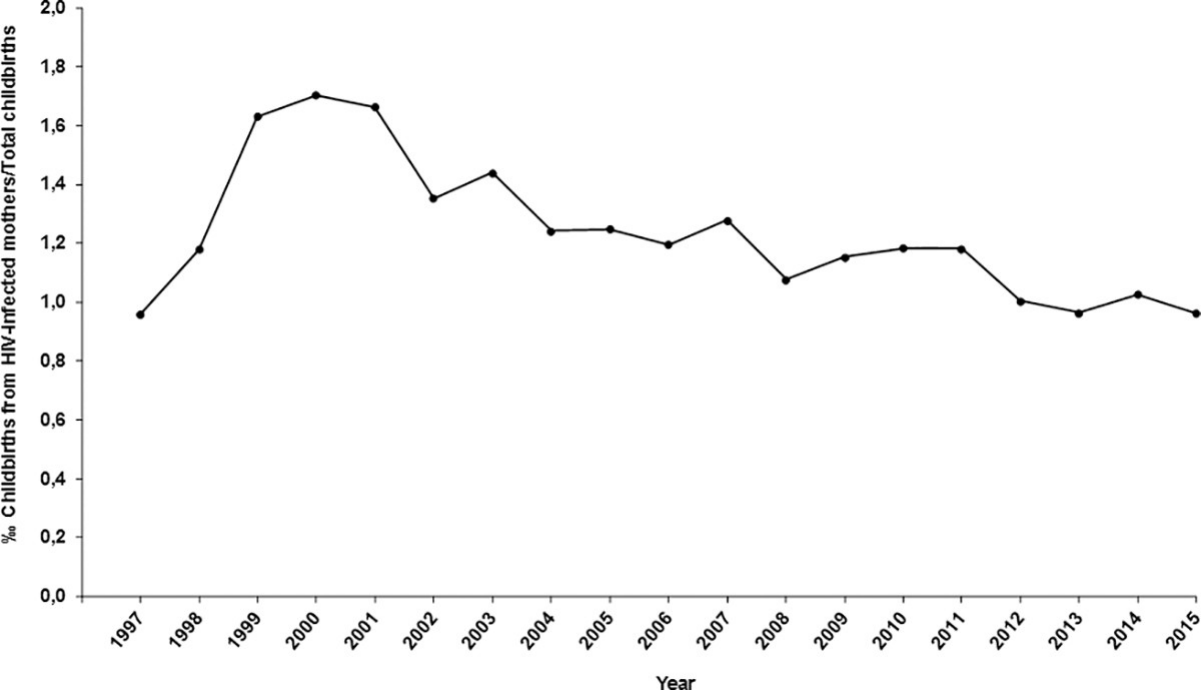
Proportion of childbirths from HIV-infected mothers in respect to total childbirths.

From 6,441,654 childbirths reviewed in MDBS, 1,364,879 (21.2%) were excluded after being adjusted by postal codes of patient’s place of residence. Of the remaining 5,076,775 childbirths, 6328 were from HIV-infected mothers (1.25‰). The proportion of childbirths from HIV-infected mothers respect to total childbirths increased from 0.96‰ in 1997 to 1.7‰ in 2000 and then decreased to 0.96‰ in 2015 (Fig 3).

**Fig 4:**
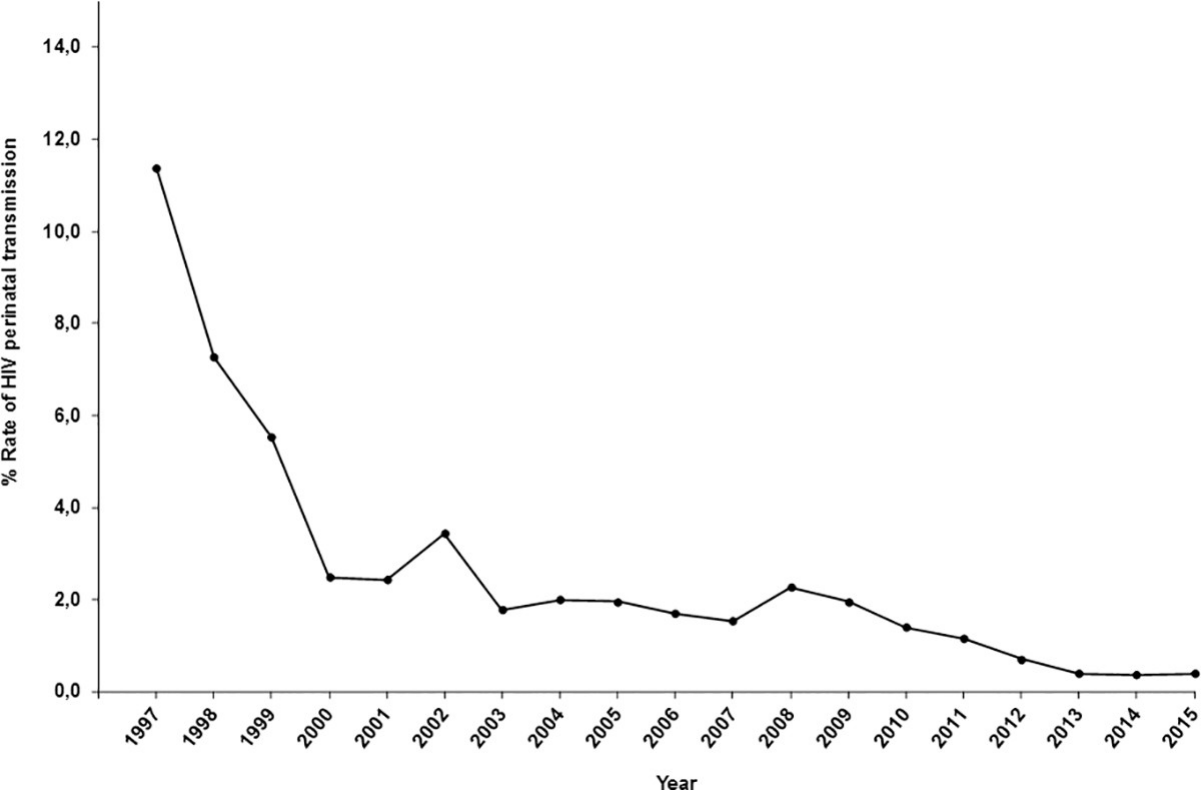
Rate of perinatal HIV transmission from 1997 to 2015.

From 387 perinatally HIV-infected children born in Spain, 308 (79.6%) were born from 1st January 1997 to 31st December 2015. Of them, 25 (8.1%) were excluded to adjust the number of childbirths by postal codes of patient’s place of residence. Of the remaining 283 children, in 158 (55.8%) their mothers were diagnosed until childbirth (Fig 1). The estimated rate of perinatal HIV transmission in Spain decreased from 11.4% in 1997 to 0.4% in 2015 (Fig 4).

## Discussion

This is the first nationwide study showing the changes in characteristics of HIV-infected children and the trends of perinatal HIV transmission in Spain in the era of cART [6–9]. In our study 532 children, who were HIV-diagnosed in Spain between 1997 and 2015, were included. We observed a decrease in the number of HIV diagnoses throughout the study period, mainly in Spanish HIV-infected children, and an increase in the number of HIV diagnoses in teen-agers with sexual infection. We also observed a decrease in the rates of new perinatal HIV diagnoses and perinatal transmission in children born in Spain.

Of the 532 children included, 406 were Spanish (76.3%) and 126 were immigrants (23.7%). Immigrant children came mainly from sub-Saharan Africa, as other studies made in Western Europe [4, 18–19], and Latin America. These children probably immigrated to Spain with their families for linguistic and cultural reasons [20]. The immigrant children had worse clinical and immunological situation at HIV diagnosis than Spanish children. It is essential to screen immigrant children, who emigrate from countries with high HIV prevalence, to achieve an earlier diagnosis and start as soon as possible cART [3].

We observed a decline in the number of new diagnoses from 1997 to 2015, mainly in Spanish children. However, the number of immigrant children increased from 1997 to 2010 and decreased until 2015. Spain was a major migrant destination during the first decade of this century, but immigration declined afterwards, probably due to the Spanish financial crisis. Therefore, the number of HIV diagnoses in immigrant children grew from 1997 to 2010 and declined until 2015 [21].

The increase in the number of HIV diagnoses in teen-agers by sexual contact is also remarkable. Teen-agers and young adults represent an increasing group of HIV-infected people worldwide. Approximately, 590,000 young adults from 15 to 24 years-old were HIV-infected worldwide in 2017. A recent study shows that young adults in Spain do not have appropriate knowledge of modes of HIV transmission [22]. Some possible reasons may include loss of fear of AIDS, motivated by the efficacy of cART, recreational drug associated with sexual intercourse or use of mobile applications to search risky sexual contacts [23, 24]. Public awareness campaigns directed to teen-agers are advisable to prevent HIV infection by sexual contact [25, 26].

Out of 387 Spanish perinatally HIV-infected children, the origin of 373 mothers was known, 299 were Spanish (80.2%) and 74 were immigrants (19.8%). Immigrant mothers came mainly from sub-Saharan Africa and Latin America. We observed a decrease in the number of perinatal HIV diagnoses throughout the study period, mainly in children born from Spanish mothers. However, the number of children born from immigrant mothers increased from 1997 to 2010 and decreased until 2015. This trend was similar to the one observed in HIV-infected immigrant children and was probably due to the Spanish financial crisis [21]. We also saw a decrease in the percentage of children with CDC B and C stage and an increase in %CD4 at diagnosis through the calendar periods. These findings were probably due to the administration of HIV testing to all pregnant women, according to the recommendations of the Spanish Health Ministry and Clinical Guidelines. Subsequently, new perinatal HIV infections were prevented, fewer HIV-infected mothers were diagnosed after childbirth and fewer HIV-infected children were diagnosed with bad clinical and immune situation [1–4, 27–28]. Interestingly, we saw a decrease in the number of IDU mothers and HCV-coinfected children. Spain was one of the European countries more affected by the heroin epidemic in the 80’s. Therefore, most of HIV and HCV co-infections during the first years of the HIV epidemic took place through intravenous drugs [29]. However, the number of IDU decreased from 1994 (5102 out of 7495 new AIDS cases (68.1%)), to 2015 (84 out of 597 new AIDS cases (14.1%)) [10].

We saw a decline in the rates of perinatal HIV diagnoses and perinatal transmission in Spanish children throughout the study period, due to PMTCT measures. However, this decline could be also influenced by the decrease observed in the number of HIV-infected mothers from 2000 to 2015. The number of new perinatal HIV diagnoses in Europe decreased by 47% from 2008 (673) to 2017 (360), representing 1.4% of all new HIV diagnoses in 2008 and 0.8% in 2017 [18]. In this sense, current rates in perinatal HIV transmission in Spain are similar to the ones observed in other European and American countries [30–35].

Furthermore, our results suggest that Spain meets the three World and Health Organization (WHO) impact targets for elimination of perinatal HIV transmission [36]. Our study shows that: 1) the number of diagnoses of Spanish perinatally HIV-infected children decreased through the calendar periods, from P1 (174) to P4 (19), whereas total childbirths ranged between 293,518 in 2011 to 262,828 in 2015; 2) the rate of perinatal HIV transmission was below 2% from 2010 (1.4%) to 2015 (0.4%). Regarding the third impact target, there is no Spanish registry of HIV-infected pregnant women. In consequence, it is not possible to know the real number of pregnant women living with HIV in Spain. However, coverage of HIV screening for pregnant women is universal in Spain. The Spanish Clinical Guides recommend HIV screening for all pregnant women within the first 3 months of pregnancy and in the third trimester if the previous test was negative. Furthermore, PMTCT measures are offered to all HIV-diagnosed pregnant women, and nucleic acid tests to detect HIV RNA or DNA are available for infant testing at birth, at 4–6 weeks and at 4–6 months. Thus, Spain would meet the three impact targets and the validation criteria for elimination of perinatal HIV transmission.

Our study has important limitations: first, SPHU from some provinces could not participate in our study. Second, the study used retrospective data, and some data of children included in our study were not available. However, the CoRISpe collects data from approximately 85% of all HIV-infected children diagnosed in Spain. Furthermore, the rates of perinatal HIV diagnoses and perinatal transmission were adjusted to consider HIV-infected children followed in SPHU not participating in the CoRISpe. Therefore, we believe that the data of SPHU children who do not participate in the CoRISpe and the missing data of the children included in the study, likely did not affect our results. Third, MDBS does not collect data about the clinical and immunological situation of HIV-infected mothers, origin, time of HIV diagnosis and cART received during their pregnancy and at childbirth. Therefore, it is not possible to know if there are any differences in regard to risk factors for perinatal HIV transmission between Spanish and immigrant mothers. Fourth, MDBS only collects data from public hospitals, and it is possible that some HIV-infected mothers gave birth in private hospitals or even at home. In consequence, the proportion of childbirths from HIV-infected mothers in respect to total childbirths and the rate of perinatal transmission could be different. Fifth, postal codes of patient’s place of residence were used to adjust the number of childbirths, and it is possible that some mothers gave birth in hospitals belonging to other provinces. Sixth, there are neither registries nor studies regarding voluntary abortions in HIV-infected women in Spain. Data from MDBS used in our study included only hospitalizations that were coded with a childbirth procedure. An epidemiological surveillance system of HIV-infected pregnant women in Spain could be set up, to clarify these questions.

In conclusion, we think that additional measures are needed to minimize the risk of perinatal HIV transmission in Spain. Universal antenatal screening is important to identify HIV-infected women. Therefore, HIV testing should be offered to all women of childbearing age and women who seek preconception counselling. In addition, routine HIV testing should be also offered to teen-agers and young adult patients in primary health care clinics [37]. Although immigrant pregnant women have universal healthcare in Spain, we think that pregnancy awareness campaigns should be organised among immigrants. Finally, an epidemiological surveillance system of HIV-infected pregnant women in Spain could be set up, to make further studies about trends and risk factors in perinatal HIV transmission.

## Supporting information

S1 Table Sociodemographic, clinical, immunological, and virological profile of the HIV-infected children at diagnosis, by origin of the children. (DOCX)

S2 Table Demographic, clinical, immunological, and virological profile of Spanish HIV-infected children at diagnosis, by origin of the mothers. (DOCX)
