# Supplementary material for: Sociodemographic changes and trends in the rates of new perinatal HIV diagnoses and transmission in Spain from 1997 to 2015
Source: PLoS One. 2019 Oct 24;14(10):e0223536. doi: 10.1371/journal.pone.0223536 (PMC6812742; doi:10.1371/journal.pone.0223536)
Supplement: S1 Table — (DOCX) [file pone.0223536.s001.docx]

**S1 Table. Sociodemographic, clinical, immunological and virological profile of the HIV-infected children at diagnosis, by origin of the children**

|  | **Spanish** | **sub-Saharan Africa** | **Latin**  **America** | **Other**  **regions** |
| --- | --- | --- | --- | --- |
|  | N=406 | N=82 | N=27 | N=17 |
| **Calendar period, N (%)** |  |  |  |  |
| 1997-2000 | 191 (47) | 6 (7.3) | 3 (11.1) | 3 (17.6) |
| 2001-2005 | 118 (29.1) | 21 (25.6) | 7 (25.9) | 3 (17.6) |
| 2006-2010 | 73 (18) | 38 (46.3) | 12 (44.4) | 7 (41.2) |
| 2011-2015 | 24 (5.9) | 17 (20.7) | 5 (18.5) | 4 (23.5) |
| **Sex, N (%)** |  |  |  |  |
| Male | 187 (46.1) | 42 (51.2) | 13 (48.1) | 9 (52.9) |
| Female | 219 (53.9) | 40 (48.8) | 14 (51.9) | 8 (47.1) |
| **HIV transmission route, N (%)** |  |  |  |  |
| Perinatal | 387 (95.3) | 61 (74.4) | 14 (51.9) | 14 (82.3) |
| Transfusional | 3 (0.7) | 8 (9.8) | 3 (11.1) | 0 (0) |
| Sexual | 6 (1.5) | 0 (0) | 8 (29.6) | 2 (11.8) |
| Unknown (mother not HIV infected) | 10 (2.5) | 2 (2.4) | 0 (0) | 0 (0) |
| Unknown (HIV mother status not known) | 0 (0) | 11 (13.4) | 2 (7.4) | 1 (5.9) |
| **CDC stage, N (%)** | N=402 | N=81 | N=27 | N=17 |
| N-A | 267 (66.4) | 37 (45.7) | 16 (59.3) | 8 (47.1) |
| B | 78 (19.4) | 21 (25.9) | 4 (14.8) | 6 (35.3) |
| C | 57 (14.2) | 23 (28.4) | 7 (25.9) | 3 (17.6) |
| **Coinfections, N (%)** |  |  |  |  |
| HCV | 12 (3) | 1 (1.2) | 0 (0) | 0 (0) |
| HBV | 0 (0) | 8 (9.8) | 0 (0) | 1 (5.9) |
| **%CD4, Median (IQR)** | N=346 | N=78 | N=26 | N=15 |
|  | 28 (15.2-40) | 17.5 (9.5-24.8) | 15.5 (5.2-23.8) | 17 (10.5-25.5) |
| **CD4/mm^3^, Median (IQR)** | N=348 | N=78 | N=26 | N=15 |
|  | 1176 (465-2185) | 609 (237-1143) | 389 (68-703) | 379 (239-747) |
| **Log Viral Load, Median (IQR)** | N=353 | N=77 | N=26 | N=15 |
|  | 5.2 (4.6-5.9) | 5.1 (4.4-5.6) | 5.1 (4.7-5.7) | 5 (4.4-5.6) |

Other regions: Eastern Europe (N=6), North Africa (N=5), Western Europe (N=3), Asia (N=3)
