# Supplementary material for: Sociodemographic changes and trends in the rates of new perinatal HIV diagnoses and transmission in Spain from 1997 to 2015
Source: PLoS One. 2019 Oct 24;14(10):e0223536. doi: 10.1371/journal.pone.0223536 (PMC6812742; doi:10.1371/journal.pone.0223536)
Supplement: S2 Table — (DOCX) [file pone.0223536.s002.docx]

**S2 Table. Sociodemographic, clinical, immunological and virological profile at diagnosis of perinatally HIV-infected children born in Spain and their mothers, , by origin of the mothers**

|  | **Spanish** | **sub-Saharan**  **Africa** | **Latin**  **America** | **Other**  **regions** |
| --- | --- | --- | --- | --- |
|  | N=299 | N=39 | N=19 | N=16 |
| **Calendar period, N (%)** |  |  |  |  |
| 1997-2000 | 163 (54.5) | 5 (12.8) | 5 (26.3) | 1 (6.3) |
| 2001-2005 | 95 (31.8) | 11 (28.2) | 5 (26.3) | 4 (25) |
| 2006-2010 | 32 (10.7) | 17 (43.6) | 5 (26.3) | 11 (68.7) |
| 2011-2015 | 9 (3) | 6 (15.4) | 4 (21.1) | 0 (0) |
| **Period of HIV diagnosis for mothers, N (%)** |  |  |  |  |
| Until childbirth | 140 (46.8) | 21 (53.8) | 10 (52.6) | 11 (68.7) |
| After childbirth | 159 (53.2) | 18 (46.2) | 9 (47.4) | 5 (31.3) |
| **Mode of HIV transmission of mothers, N (%)** | N=281 | N=38 | N=18 | N=15 |
| IDU | 146 (52) | 1 (2.6) | 2 (11.1) | 1 (6.7) |
| No history of IDU | 135 (48) | 37 (97.4) | 16 (88.9) | 14 (93.3) |
| **Sex of children, N (%)** |  |  |  |  |
| Male | 139 (46.5) | 15 (38.5) | 4 (21.1) | 10 (50) |
| Female | 160 (53.5) | 24 (61.5) | 15 (78.9) | 6 (50) |
| **Age of children, N (%)** |  |  |  |  |
| <1 year | 188 (62.9) | 27 (69.2) | 12 (63.2) | 12 (83.3) |
| 1-5 years | 76 (25.4) | 11 (28.2) | 6 (31.6) | 4 (16.7) |
| >5 years | 35 (11.7) | 1 (2.6) | 1 (5.3) | 0 (0) |
| **Coinfections in children, N (%)** |  |  |  |  |
| HCV | 10 (3.3) | 0 (0) | 1 (5.3) | 0 (0) |
| HBV | 0 (0) | 0 (0) | 0 (0) | 0 (0) |
| **CDC stage of children, N (%)** | N=295 | N=39 | N=19 | N=16 |
| N-A | 190 (64.4) | 32 (82.1) | 13 (68.4) | 14 (87.5) |
| B | 60 (20.3) | 3 (7.7) | 3 (15.8) | 1 (6.3) |
| C | 45 (15.3) | 4 (10.3) | 3 (15.8) | 1 (6.3) |
| **%CD4, Median (IQR)** | N=247 | N=35 | N=19 | N=14 |
|  | 27 (15-38.7) | 31 (21.9-44.3) | 38.3 (22-46.5) | 36 (30.4-44) |
| **CD4/mm^3^, Median (IQR)** | N=249 | N=35 | N=19 | N=14 |
|  | 1180 (465-2142) | 1518 (915-2885) | 1928 (638-2239) | 2119 (972-3068) |
| **Log Viral Load, Median (IQR)** | N=256 | N=35 | N=17 | N=13 |
|  | 5.4 (4.8-5.9) | 4.8 (4-5.9) | 5.2 (4.4-5.9) | 5.7 (5.1-5.9) |

Other regions: Eastern Europe (N=6), North Africa (N=6), Western Europe (N=4)
